# Size-Tunable Natural Mineral-Molybdenite for Lithium-Ion Batteries Toward: Enhanced Storage Capacity and Quicken Ions Transferring

**DOI:** 10.3389/fchem.2018.00389

**Published:** 2018-08-28

**Authors:** Feng Jiang, Sijie Li, Peng Ge, Honghu Tang, Sultan A. Khoso, Chenyang Zhang, Yue Yang, Hongshuai Hou, Yuehua Hu, Wei Sun, Xiaobo Ji

**Affiliations:** ^1^School of Minerals Processing and Bioengineering, Central South University, Changsha, China; ^2^College of Chemistry and Chemical Engineering, Central South University, Changsha, China; ^3^State Key Laboratory of Powder Metallurgy, Central South University, Changsha, China

**Keywords:** natural molybdenite ore, molybdenum disulfide, size effect, lithium-ion battery, electrochemical performance

## Abstract

Restricted by the dissatisfied capacity of traditional materials, lithium-ion batteries (LIBs) still suffer from the low energy-density. The pursuing of natural electrode resources with high lithium-storage capability has triggered a plenty of activities. Through the hydro-refining process of raw molybdenite ore, containing crushing–grinding, flotation, exfoliation, and gradient centrifugation, 2D molybdenum disulfide (MoS_2_) with high purity is massively obtained. The effective tailoring process further induce various sizes (5, 2, 1 and 90 nm) of sheets, accompanying with the increasing of active sites and defects. Utilized as LIB anodes, size-tuning could serve crucial roles on the electrochemical properties. Among them, MoS_2_-1 μm delivers an initial charge capacity of 904 mAh g^−1^, reaching up to 1,337 mAh g^−1^ over 125 loops at 0.1 A g^−1^. Even at 5.0 A g^−1^, a considerable capacity of 682 mAh g^−1^ is remained. Detailedly analyzing kinetic origins reveals that size-controlling would bring about lowered charge transfer resistance and quicken ions diffusion. The work is anticipated to shed light on the effect of different MoS_2_ sheet sizes on Li-capacity ability and provides a promising strategy for the commercial-scale production of natural mineral as high-capacity anodes.

## Introduction

Lithium-ion batteries (LIBs) are renewable energy storage devices commonly used in consumer electronics, high-power tools, and electric vehicles because of their excellent capacities, such as high energy density, long cycle life, low self-discharge, no memory effect (Li et al., 2017; Yang et al., 2017; Zhang et al., 2018; Zheng et al., 2018). Graphite is the current commercial anode material due to its flat potential profile and great structure stability during cycling. However, six carbon atoms are required to accommodate one Li ion, and the theoretical specific capacity (372 mAh g^−1^) of graphite is insufficient to meet the increasing requirements of the ever-growing market of high-performance batteries(Shim and Striebel, 2003; Yoshio et al., 2003, 2004).

Two-dimensional (2D) metal dichalcogenides (MDCs) as an alternative material for graphite has received considerable attention(Chhowalla et al., 2013; Yang et al., 2015; Zhang et al., 2015; Ge et al., 2018a,b). Among them, molybdenum disulfide is a typical graphene analog, in which two adjacent S-Mo-S layers are linked by weak van der Waals forces. Given its novel mechanical, optical, electrical, and electrochemical properties, MoS_2_ has been widely studied for different applications in lubricants(Xiao et al., 2017; Wu et al., 2018), photocatalytic degradation catalysts(Li et al., 2014; Su et al., 2016; Liu et al., 2018), sensors(Liu et al., 2014; Wang and Ni, 2014), electrocatalytic hydrogen generation(Gao et al., 2015a,b; Zhu et al., 2015; Geng et al., 2016), field-effect transistors(Dankert et al., 2014; Roy et al., 2014), supercapacitors(Ma et al., 2013; Acerce et al., 2015), and electrode material for batteries(Liang et al., 2011; Yang et al., 2015; Hai et al., 2018). Compared with graphite, MoS_2_ has a wider lattice spacing (~0.65 nm), which is conducive to rapid insertion and extraction of alkali metal ions. After insertion, Li_x_MoS_2_ can further react with Li^+^ ions to form Li_2_S and Mo atom, and the theoretical specific capacity of MoS_2_ in LIBs is 670 mAh g^−1^, which is much higher than that of graphite (Stephenson et al., 2014). Meanwhile, a number of studies reported that the capacity of MoS_2_ can reach >1,000 mAh g^−1^, which arises from Mo atoms accommodating a large amount of Li ions over prolonged discharging process (Wang et al., 2018).

Most previous studies synthesized MoS_2_ by chemical methods to obtain nanosheets with desired size and thickness. Hydrothermal, chemical vapor deposition, and hot injection are typical approaches that use molybdenum salts as precursors (Altavilla et al., 2011; Wang et al., 2014). Although the aforementioned chemical synthetic methods can be used for the large-scale preparation of MoS_2_ nanosheets, their industrial applications are limited by their rigid reaction conditions and environmentally pernicious reactants (Yang et al., 2016, 2018; Zhang et al., 2016). MoS_2_ is abundant in the form of molybdenite in nature and is generally extracted and processed into molybdenum metal and compounds through beneficiation, smelting and chemical synthesis. Thus, fabrication of MoS_2_ materials directly from natural molybdenite ore can eliminate many intermedia complex processes and reduce synthetic contaminants. In addition, the appropriate size of MoS_2_ for LIBs remains unknown. In view of the fact that size exerts a noteworthy influence on the electrochemical properties of many materials (Kim et al., 2005; Liu et al., 2005; Drezen et al., 2007; Wagemaker et al., 2007; Kiani et al., 2010; Jiang et al., 2017), understanding the effects of different sizes of MoS_2_ on battery performance and electrochemical properties is important to application of MoS_2_ in LIBs.

Herein, a hydro-refining technology combining crushing-grinding, flotation, mechanical exfoliation, and classification processes was developed to prepare a series of size-controlled MoS_2_ sheets directly from natural raw molybdenite ore. This method is simple, eco-friendly, and high-yielding. When used the as-prepared MoS_2_ sheets as LIB anodes, size displays an important effect on electrochemical properties. Among them, the MoS_2_-1 μm electrode demonstrated excellent electrochemical properties with lower charge transfer resistance and rapider Li ions diffusion, delivering a higher specific capacity and initial coulombic efficiency. These results suggest the proper MoS_2_ sheet size for LIBs and indicate the present approach is promising for industrial-scale production of natural molybdenite as high-capacity anodes.

## Materials and methods

### Materials

Natural raw ore (rock size: 5–10 cm, MoS_2_ content: 1–2%) was received from China Molybdenum Co., Ltd. Raw ore was crushed to small stones (particle size ~2 mm) and then ball-milled with water at a concentration of 66.6% to reduce the granularity. Ball-milled production, which is also called pulp (particle size: 75% <74 μm), was transferred to flotation cell, and water was added to adjust the concentration to 33%. In brief, 333 mg/L sodium silicate as depressant, 35 mg/L kerosene as molybdenite collector, and 15 mg/L terpineol as foaming agent were added sequentially to the pulp during agitation. Then, the pulp was aerated, and flotation froth was generated above the pulp and collected as the rough molybdenite concentrate (MoS_2_ content: 2–5%), which then was reground to a fineness of 85% <37 μm by stirred mill. Finally, the reground rough concentrate was flotation cleaned eight times to improve the molybdenite concentrate grade. In the first cleaning operation, 2 g/L sodium sulfide was added to the pulp as the other sulfide minerals' depressant. Then, the obtained concentrate froth was transferred to the next cleaning operation, in which the sodium sulfide dosage was half of that used in the previous step. The final concentrate froth from the eighth cleaning operation was filtered and dried to achieve molybdenite concentrate (MoS_2_ content: ~92%).

Differently sized MoS_2_ sheets were prepared through an intense shearing process. Molybdenite concentrate (10 g), polyvinylpyrrolidone-K30 (0.25 g, PVP-K30), and deionized water (500 mL) were placed in a stainless steel homogenizer. The homogenizer was run at 12,000 rpm for 5 h to exfoliate the molybdenite content and acquire a MoS_2_ suspension. The homogeneous dispersion was gradient centrifuged at 1,000, 3,000, 5,500, and 10,000 rpm, and the precipitates were collected and rinsed by deionized water several times to remove the residual PVP. Afterward, the as-prepared differently sized MoS_2_ sheets were dried at 60°C in a vacuum oven for 24 h.

### Material characterization

The crystal structure of the as-prepared materials was identified by X-ray diffraction (XRD, Bruker D8 diffractometer with monochromatic Cu Kα radiation and wavelength of 1.5406 Å). The composition of the samples was characterized by X-ray fluorescence (XRF). The particle size distribution was measured by laser diffraction (Malvern Mastersizer 2000). The morphology was analyzed by field emission scanning electron microscopy (FEI Quanta 200, Japan) and atomic force microscopy (AFM, Bruker Multimode V, Germany).

### Electrochemical characterization

The active materials, carboxymethyl cellulose, and conductive additive (Super P, carbon black) were mixed in a weight ratio of 75:15:15 by using deionized water as the solvent. Then, the steady slurry was evenly painted on a copper foil. After drying at 80°C in a vacuum oven for 12 h, the copper foil was cut into wafer electrodes. The mass of the active material in each electrode was approximately 1.0 mg cm^−2^. The CR2016 coin-type cells were assembled in an argon-filled glovebox (MBRAUN, Germany) by using as-prepared electrodes as the anode, metallic lithium disk as the counter electrode, and LiClO_4_ (1 M) in ethylene carbonate and dimethyl carbonate (1:1, v/v) as the electrolyte. The capacities of Li-ion half cells were measured at different current densities in the voltage range of 0.01–3 V vs. Li^+^/Li by using an Arbin battery testing system (BT2000). Cyclic voltammetry (CV) was performed by CHI660D electrochemical station (Shanghai Chenhua, China) in the voltage range of 0.01–3 V vs. Li^+^/Li. Electrochemical impedance spectroscopy (EIS) was performed at the frequency range of 0.01 Hz to 100 kHz, and the excitation amplitude applied to the cells was 5 mV. All of the electrochemical tests were conducted at a temperature of 25°C.

## Results and discussion

**Figure 1:**
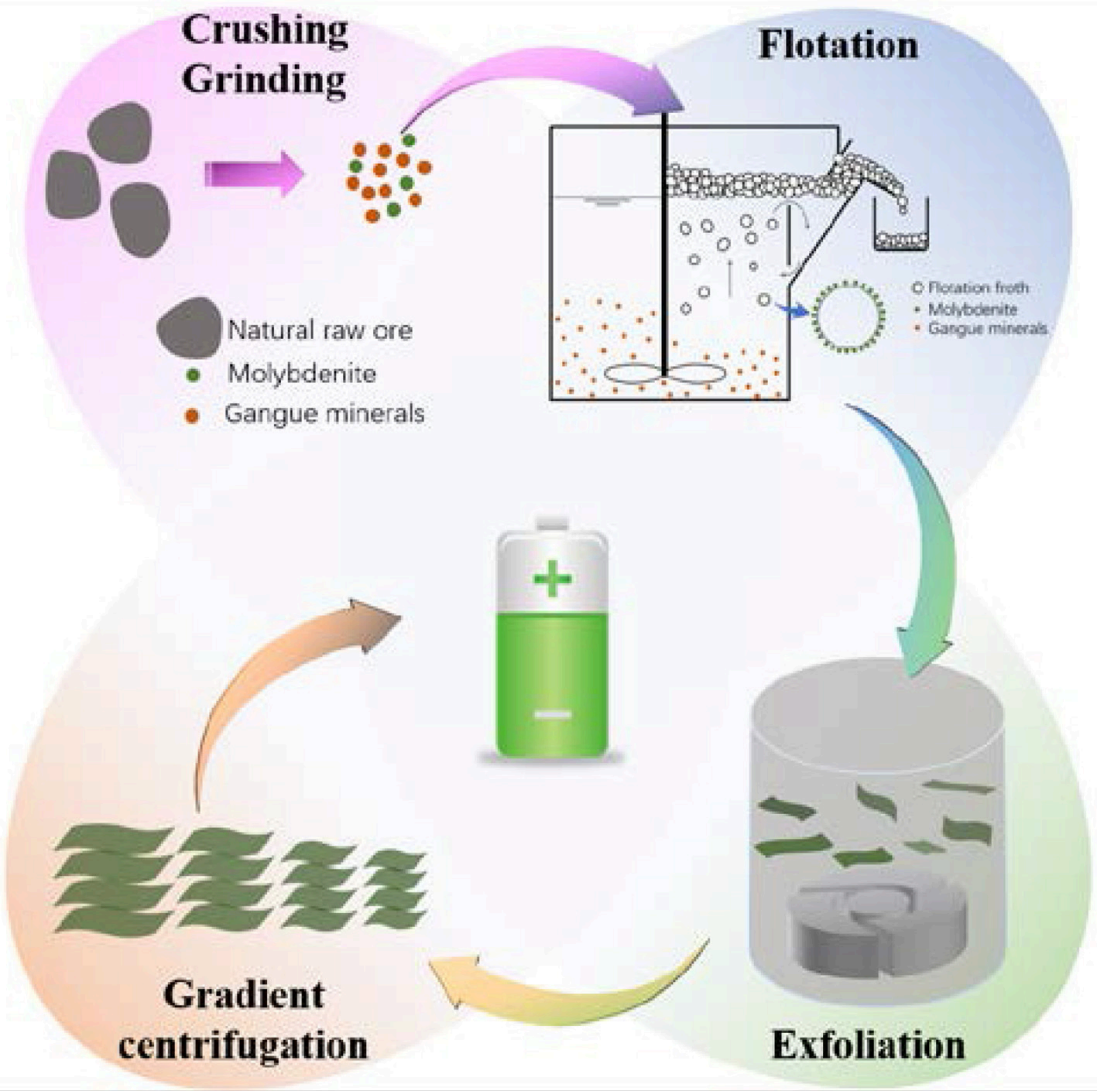
Schematic plan of hydro-refining process of natural raw ore.

A schematic showing the hydro-refining process of preparing a series of size-controlled MoS_2_ sheets directly from natural raw ore is illustrated in Figure 1. Initially, the particle size of natural raw ore is reduced by crushing and ball milling. Using flotation, molybdenite in the form of concentrate froth is separated from other nontarget minerals, and the recovery rate of molybdenite is ~85%. The obtained molybdenite concentrate is further downsized by a homogenizer, which has a strong shearing force to exfoliate bulky molybdenite (i.e., MoS_2_). Finally, the MoS_2_ suspension is size-classified via high-speed gradient centrifugation. This method is low cost, environmental friendly, high-yielding, and is very promising for the large-scale preparation of MoS_2_ sheets with various sizes.

**Figure 2:**
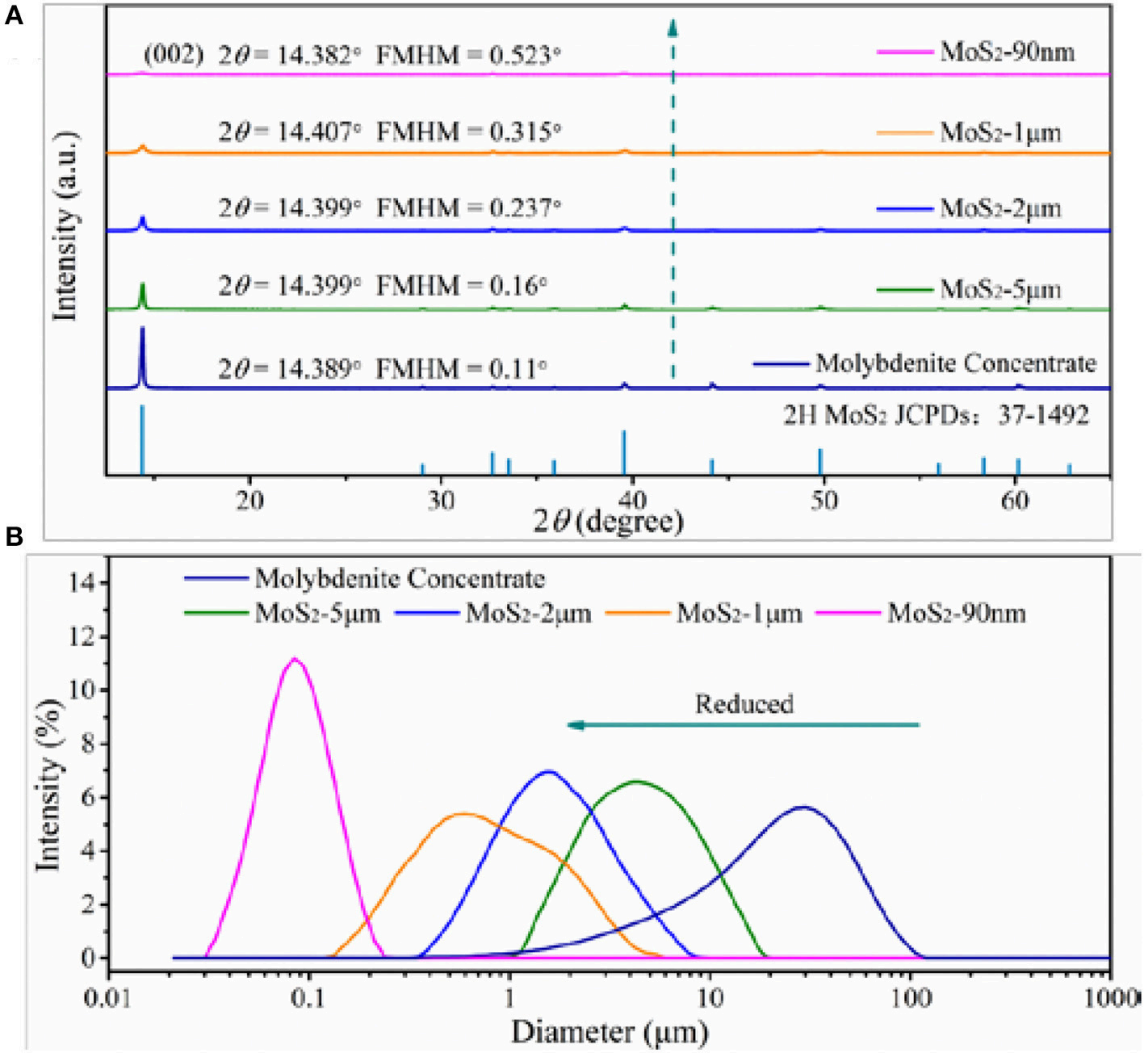
**(A)** XRD spectra of molybdenite concentrate and MoS_2_ samples. **(B)** Particle size analysis of molybdenite concentrate and MoS_2_ samples.

The chemical composition of the natural raw ore and molybdenite concentrate is presented in Table 1. In natural raw ore, the dominant elements are O and Si, while the Mo content is only 0.82%, thus a facile and low-cost flotation process is indispensable to obtain pure molybdenite concentrate (Jiangang et al., 2012; Liu et al., 2012a). After flotation, the Mo content can reach to 55%, representing the high purity of the molybdenite concentrate. The slight oxidation of the natural molybdenite surface is due to the exposure to oxidative environment. The crystal structures and phases of the molybdenite concentrate and the differently sized MoS_2_ are investigated by XRD (Figure 2A). All of these samples exhibit similar XRD patterns, which match well with the 2H MoS_2_ phase (JCPDS no. 37-1492) (Ding et al., 2012; Xie et al., 2015; Sun et al., 2017). No extra peaks appear in the pattern, indicating their high purity, which agrees well with the XRF results. The peak at approximately 14.4° is the characteristic peak of (002) facet. Decreasing peak intensity and broadening peak width of (002) facet signify the thickness reduction of MoS_2_ sheets (Wang et al., 2013d). Using the results from the XRD patterns, we calculate the grain parameters of each sample by the Scherrer equation:

(1) $$\begin{array}{l} D=K\lambda /\beta cos\theta \end{array}$$

Where, *D* is the grain size, *K* is the Scherrer constant (0.89), λ is the diffraction light (X-ray) wavelength (0.15406 nm), β is the full width at half maximum, and θ is the Bragg angle. As shown in Table 2, the MoS_2_-90 nm sample has the smallest grain size among them. Moreover, the volume average diameters of the samples are tested with a laser diffraction-based particle size analyzer. As shown in Figure 2B, the volume average diameters of the molybdenite concentrate and differently sized MoS_2_ are 25.964, 5.346, 1.978, 1.023, and 0.092 μm, respectively.

**Table 1:** Chemical composition of natural raw ore and molybdenite concentrate.

**Element (wt %)**	**Mo**	**S**	**O**	**Fe**	**Si**
Natural raw ore	0.82	0.48	46.21	1.56	30.66
Molybdenite concentrate	55.61	40.00	2.140	0.15	0.016

**Table 2:** Crystal parameters of molybdenite concentrate and MoS_2_ samples.

	**Molybdenite concentrate**	**MoS_2_-5 μm**	**MoS_2_-2 μm**	**MoS_2_-1 μm**	**MoS_2_-90 nm**
2θ (deg)	13.389	14.399	14.399	14.407	14.382
β (rad)	0.0019	0.0028	0.0041	0.0055	0.0091
*D* (nm)	70.34	48.36	32.65	24.56	14.79

**Figure 3:**
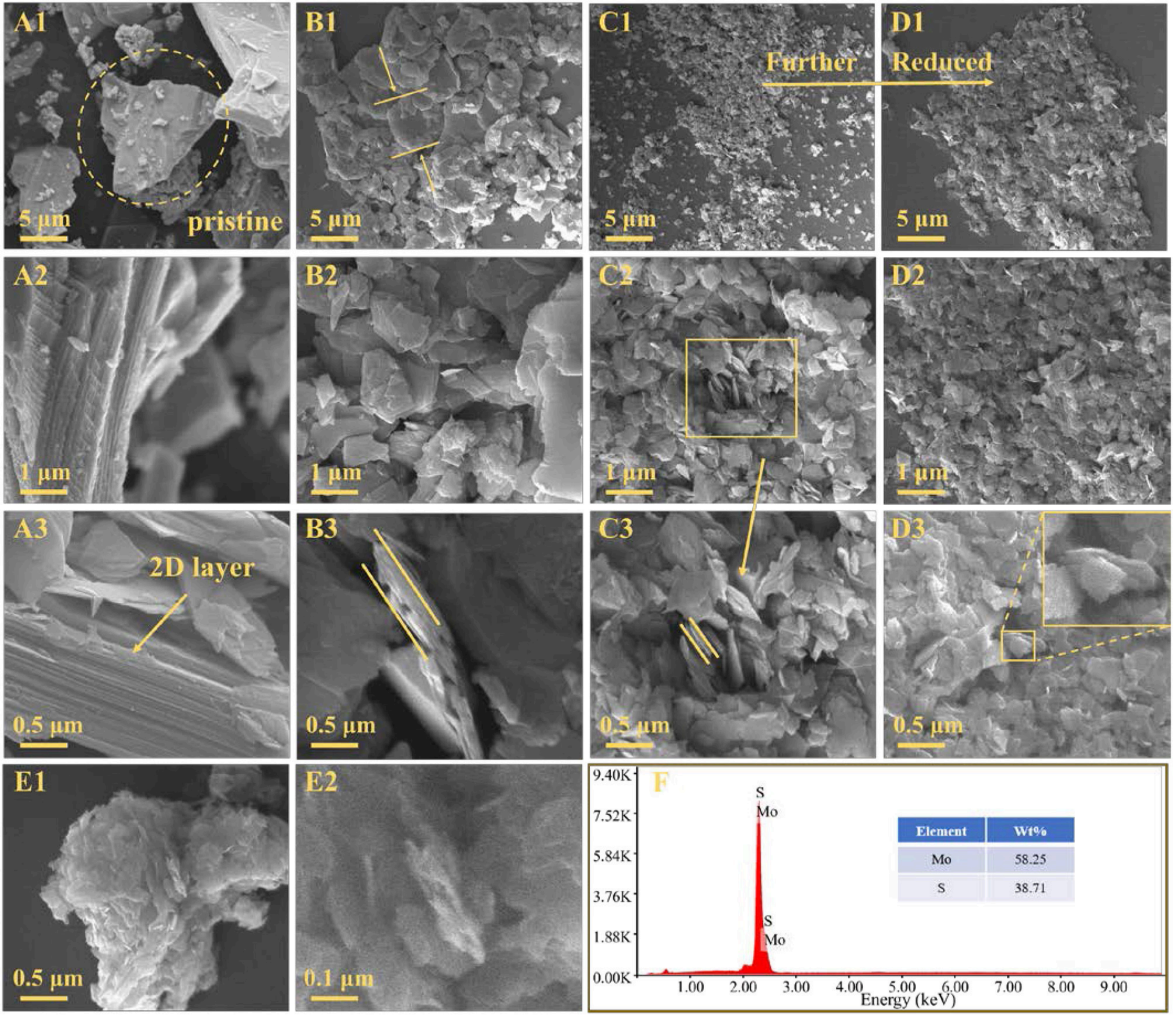
SEM images of **(A1–A3)** molybdenite concentrate, **(B1–B3)** MoS_2_-5 μm, **(C1–C3)** MoS_2_-2 μm, **(D1–D3)** MoS_2_-1 μm and **(E1, E2)** MoS_2_-90 nm. **(F)** Energy-dispersive spectrometry (EDS) of molybdenite concentrate.

The morphological of the samples are conducted by SEM and shown in Figure 3. Figure 3A1 shows the morphology of the molybdenite concentrate where molybdenite particles exhibit various textures (flaky, blocky, and irregular shapes), and their size is mainly tens of microns, which can be attributed to the complex factors in natural mineralization. In addition, several small pieces of debris are found on the surface of large molybdenite particles with a size distribution from a few microns to submicron. From the higher-magnification observations, stacked compacted 2D layer structure is found distinctly in Figures 3A2,A3. By contrast, MoS_2_-5 μm, MoS_2_-2 μm, and MoS_2_-1 μm show a lamellar morphology. As shown in Figures 3B1–B3, several thick sheets with size of ~5 μm are distributed in the MoS_2_-5 μm sample, which thickness is around 300 nm. Meanwhile, stratified structure and uneven edges are detected, accompanying with an increasing of active sites and defects. In the SEM images of MoS_2_-2 μm and MoS_2_-1 μm, small sheets with average sizes of ~1 μm and ~500 nm can be observed. The curved sheets shown in the higher-magnification images of Figures 3C3,D3 indicate the thinness and flexibility of the MoS_2_ sheets, which significantly ease the volume expansion during the charge and discharge cycles and enhance the stability of the batteries. Figures 3E1,E2 show the compact agglomeration of nano-MoS_2_ sheets in the MoS_2_-90 nm sample, revealing the strong tendency of MoS_2_ nanosheets to aggregate because of their high surface area and energy. This agglomeration dramatically decreases the active sites of the material and hinder Li^+^ diffusion, which led to a low capacity. Figure 3F displays the compositions of molybdenite concentrate by energy disperse spectroscopy (EDS) analysis. No evident incidental element appears, and the atomic ratio of S to Mo is approximately 2, which further demonstrate the high purity of the molybdenite concentrate obtained from natural raw ore.

For exploring the crystalline characteristics of MoS_2_ sample, TEM and HRTEM tests with various magnifications are performed. As shown in Figures S1A,B, thin sheets are detected in MoS_2_-1 μm, accompanying with clear 2D layer structure. Figure S1C shows the HRTEM image of MoS_2_-1 μm, revealing the abundant defects existing in MoS_2_ sheets. Stripes spaced 0.273 nm apart in the insetmap are in good accordance with the (100) facet of MoS_2_, as well as the single-crystal SAED pattern of MoS_2_-1 μm shows the typical hexagonal spot pattern (Figure S1D). AFM tests are further carried out to obtain detailed information about the morphologies of MoS_2_-5 μm, MoS_2_-2 μm, MoS_2_-1 μm, and MoS_2_-90 nm. As shown in Figures S2A2–C2, the thickness of the MoS_2_ sheets in the MoS_2_-5 μm, MoS_2_-2 μm, and MoS_2_-1 μm samples gradually decrease from ~330 to ~170 nm and then to ~100 nm. The same trend is observed for the sheet diameter (Figures S2A1–C1). Stratified structures and rough edges can also be observed in the 3D plots (Figures S2A3–C3), indicating more active sites can be exposed for Li ions. The image of the MoS_2_-90 nm sample shown in Figure S2D1 displays three irregular particles with a thickness of ~230 nm and a diameter of ~1 μm. Similar to the SEM results, the AFM findings indicate that these uncommon particles are the agglomeration of nano-MoS_2_ sheets. When observing at a small height scale, two pieces of thin films are visible with a thickness of ~0.65 nm, indicating single-layer MoS_2_ films distributing in the MoS_2_-90 nm sample.

**Figure 4:**
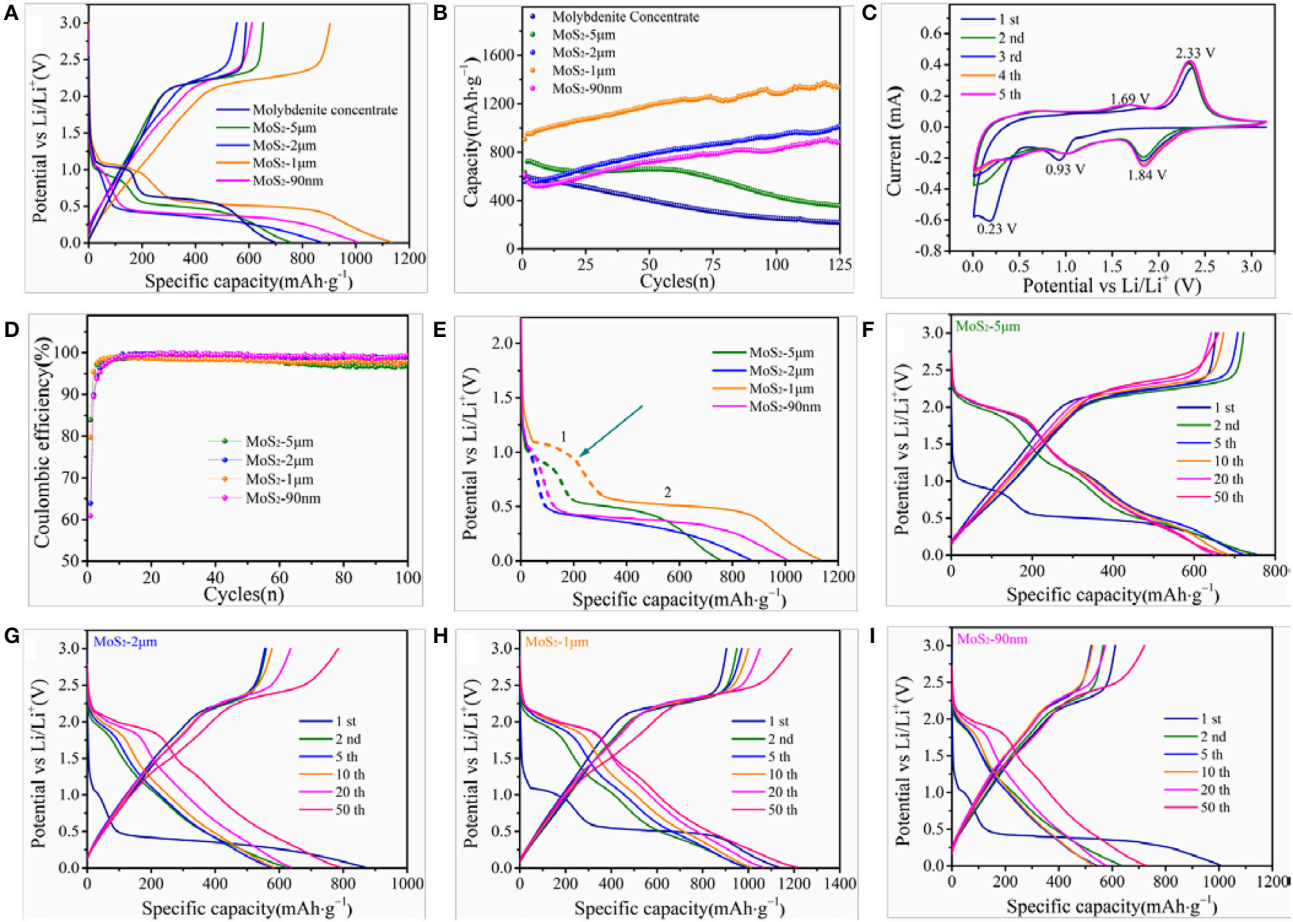
**(A)** Initial charge and discharge curves of molybdenite concentrate and MoS_2_ samples at 100 mA g^−1^. **(B)** CV curves of the MoS_2_-1μm electrode for the initial 5 cycles at a scanning rate of 0.2 mV s^−1^. **(C)** Cycling performance of molybdenite concentrate and MoS_2_ samples at 100 mA g^−1^. **(D)** Coulombic efficiency of MoS_2_ samples at 100 mA g^−1^. **(E)** Discharge curves of MoS_2_ samples in the first cycle. Galvanostatic charge and discharge profiles of **(F)** MoS_2_-5 μm, **(G)** MoS_2_-2 μm, **(H)** MoS_2_-1 μm and **(I)** MoS_2_-90 nm electrodes at 100 mA g^−1^.

The electrochemical properties of the as-prepared samples are measured by galvanostatic charge–discharge test at various current densities. Figure 4A shows the initial charge and discharge curves of molybdenite concentrate and MoS_2_ samples at 100 mA g^−1^, where two potential plateaus at approximately 1.1 and 0.6 V vs. Li/Li^+^ in the first discharge (lithiation) of the electrodes are observed. The first plateau at 1.1 V could be attributed to the intercalation of Li^+^ into MoS_2_ interlayers (MoS_2_ + *x*Li^+^ + *x*e^−^ → Li_*x*_MoS_2_), and the low plateau at 0.6 V is due to the conversion reaction of Li_*x*_MoS_2_ to Mo metal and Li_2_S (Li_*x*_MoS_2_ + (4 – *x*)Li^+^ + (4 – *x*)e^−^ → Mo + 2Li_2_S). Only one significant potential plateau at approximately 2.3 V appeared in the first charge (delithiation) process, and it corresponds to the delithiation of Li_2_S (Li_2_S – 2e^−^ → 2Li^+^ + S). This result demonstrates that the conversion reaction is irreversible (Xiao et al., 2010; Stephenson et al., 2014). The electrochemical behavior is further analyzed by CV (Figure 4B). In the first cathodic sweep, two peaks appear at approximately 0.93 and 0.23 V, which are attributed to the insertion and conversion reactions, respectively. Meanwhile, these two peaks weaken in subsequent cathodic cycles. Instead, a sharp reduction peak arises at approximately 1.84 V, which matches well with the behavior in Li-S battery and corresponds to the reaction of S to Li_2_S (Ji and Nazar, 2010; Elazari et al., 2011). In the anodic sweep, one shallow peak at 1.69 V and one sharp peak at 2.33 V are observed. The first oxidation peak is due to the delithiation of residual Li_*x*_MoS_2_, and the latter peak represents the conversion of Li_2_S to S (Song et al., 2013; Stephenson et al., 2014).

As shown in Figure 4A, the initial discharge specific capacities of molybdenite concentrate, MoS_2_-5 μm, MoS_2_-2 μm, MoS_2_-1 μm, and MoS_2_-90 nm are 688, 779, 868, 1134, and 1004 mAh g^−1^ at 100 mA g^−1^, respectively, while the initial charge capacities are 589, 653, 555, 904, and 611 mAh g^−1^. Among them, the MoS_2_-1 μm has a higher capacity owing to its compared richer active sites. Figure 4C shows the cycling performance of the molybdenite concentrate and MoS_2_ samples at 100 mA g^−1^. The molybdenite concentrate exhibits an unsatisfied stability, which capacity gradually decreases to 217 mAh g^−1^ after 125 cycles, showing a low capacity retention of 37%. MoS_2_-5 μm displays a specific capacity of ~600 mAh g^−1^ before 50 cycles with no evident fading, while fades quickly to 355 mAh g^−1^. The poor cycling stabilities of the molybdenite concentrate and MoS_2_-5 μm can be ascribed to the large volume expansion of bulk MoS_2_ during repeated charge/discharge processes, causing the harmful shedding of active materials. Meanwhile, MoS_2_-2 μm, MoS_2_-1 μm, and MoS_2_-90 nm show excellent stability without any capacity decay. As shown, the capacities of MoS_2_-2 μm, MoS_2_-1 μm, and MoS_2_-90 nm increase with the cycling going on, reaching up to 1013, 1337, and 881 mAh g^−1^ after 125 cycles. The data reported here are higher than most of the reported works (Table 3). The promotion in capacity may be attributed to the increased Mo atoms created by the irreversible redox reaction during repeated charge/discharge processes, bringing about better conductivity. Meanwhile, Mo atoms accommodate a large amount of Li ions over prolonged discharging process, increasing the electrode's Li-capacity. The significant differences between these prepared samples indicate that decreasing the particle size of MoS_2_ can significantly improve the cycling stability and capacity of batteries due to the stronger and more flexible structure and more active spots. However, MoS_2_-90 nm displays a lower capacity than MoS_2_-1 μm may due to the particle agglomeration, accompanying with the reduction in active spots.

**Table 3:** Composition of this work and other previous reported results.

**Electrode material**	**Method**	**Morphology**	**Reserved capacity (mAh g^−1^) after (Y) cycles at (Z) current density**	**References**
MoS_2_	Hydrothermal	Nanoflakes	780 (40) (0.04 A g^−1^)	Feng et al., 2009
MoS_2_	Impregnation	Wire-like Arrays	876 (100) (0.1 A g^−1^)	Liu et al., 2012b
MoS_2_	Hydrothermal	3D Flower-like Spheres	947 (50) (0.1 A g^−1^)	Yang et al., 2014
MoS_2_	Solution Process	Restacked Nanosheets	750 (50) (0.05 A g^−1^)	Du et al., 2010
MoS_2_	Hydrothermal	Hollow Nanoparticles	902 (80) (0.1 A g^−1^)	Wang et al., 2013c
Molybdenite	Hydro-refining	**Nanosheets**	**1337 (125) (0.1 A g**^−1^**)**	**This work**

Figure 4D shows the coulombic efficiencies of the MoS_2_ samples at 100 mA g^−1^. The initial coulombic efficiencies of MoS_2_-5 μm, MoS_2_-2 μm, MoS_2_-1 μm, and MoS_2_-90 m are 83.9, 63.9, 79.7, and 60.9%, respectively, which rapidly increase to >97% after five cycles. The significant difference in initial coulombic efficiency between MoS_2_ samples can be explained through the electrochemical behavior during the first lithiation process. Unlike the conversion reaction, Li ion intercalation is a reversible reaction. Thus, a high ratio of intercalation capacity can result in a high initial coulomb efficiency. As shown in Figure 4E, the intercalation capacity ratios of MoS_2_-5 μm, MoS_2_-2 μm, MoS_2_-1 μm, and MoS_2_-90 m are calculated to be 23.06, 9.06, 23.05, and 8.26%, respectively, which correspond well to the initial coulombic efficiencies. Moreover, the initial coulombic efficiency is an important parameter that determines the industrial application feasibility of electrode materials. Individual MoS_2_-5 μm and MoS_2_-1 μm have much higher initial coulombic efficiencies, suggesting that they are more conducive to the application of full batteries than MoS_2_-2 μm and MoS_2_-90 nm.

The galvanostatic charge and discharge profiles of four MoS_2_ electrodes at 100 mA g^−1^ are shown in Figures 4F–I. Figure 4H shows that, different from the initial discharge curve, a new potential plateau emerges at 2.0 V vs. Li/Li^+^, and the two aforementioned potential plateaus at 1.1 and 0.6 V disappear in the second discharge profile. This appearance indicates that the dominant reaction of the discharge process turns into S lithiation (S + 2Li^+^ + 2e^−^ → Li_2_S) (Chang et al., 2013; Zhu et al., 2014), which is in good accordance with the aforementioned CV results. Figure 4F,G,I show the charge and discharge curves of the three other electrodes, which are similar to that of the MoS_2_-1 μm electrode.

**Figure 5:**
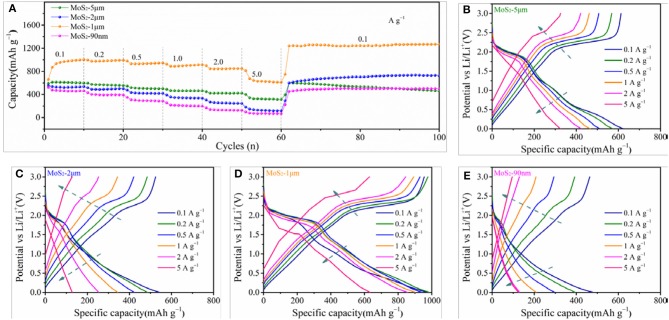
**(A)** Rate performance of MoS_2_ samples at various current densities. Comparation of the charge and discharge curves of **(B)** MoS_2_-5 μm, **(C)** MoS_2_-2 μm, **(D)** MoS_2_-1 μm, and **(E)** MoS_2_-90 nm at various current densities.

Figure 5A shows the rate performances of MoS_2_-5μm, MoS_2_-2μm, MoS_2_-1μm, and MoS_2_-90nm. Apparently, the capacity of MoS_2_-1μm is much higher than those of MoS_2_-5μm, MoS_2_-2μm, and MoS_2_-90nm. The charge capacities of the MoS_2_-1μm anode at 0.5, 1.0, 2.0, and 5.0 A g^−1^ are 931, 900, 857, and 682 mAh g^−1^, respectively. When the current density reverts to 0.1 A g^−1^, the capacity recovers to a high value of 1,239 mAh g^−1^, indicating the strong tolerance of the electrode for the rapid charge–discharge process and the remarkable capacity recoverability of the MoS_2_-1 μm electrode. Meanwhile, the charge capacities of MoS_2_-5 μm are 516, 464, and 342 mAh g^−1^ at 0.5, 1.0, 2.0, and 5.0 A g^−1^, respectively, and then reverts to 597 mAh g^−1^ at 0.1 A g^−1^, which is close to the initial capacity. However, along with increasing loops, the capacity declines following a similar pattern to the previous result. The charge capacities of MoS_2_-2 μm and MoS_2_-90 nm are 355 and 217 mAh g^−1^ at 1.0 A g^−1^ and 146 and 76 mAh g^−1^ at 5.0 A g^−1^, respectively, which are unsatisfactory. Figures 5B–E display the comparation of the charge and discharge curves of MoS_2_-5 μm, MoS_2_-2 μm, MoS_2_-1 μm, and MoS_2_-90 nm at various current densities. As shown in Figure 5E, the MoS_2_-1 μm electrode keeps a similar charge and discharge curves even at a high current density, as well as considerable capacity retention, further revealing its excellent rate performance. While for MoS_2_-5 μm, MoS_2_-2 μm, and MoS_2_-90 nm, it is difficult for them to maintain the original charge and discharge behavior at high current densities, leading to a sharp declining in capacity (Figures 5B,C,F).

**Figure 6:**
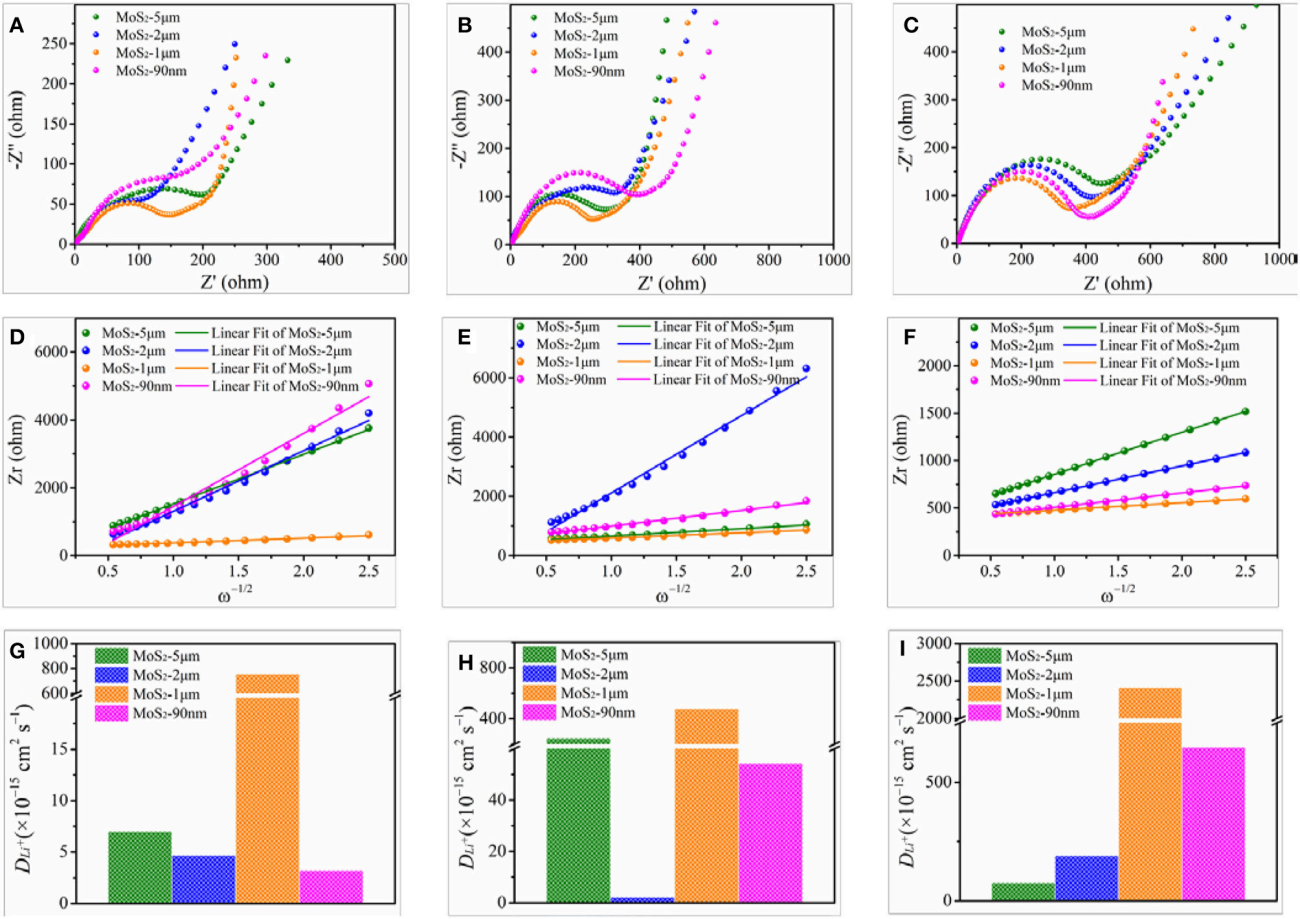
EIS curves of MoS_2_ samples at **(A)** fully uncharged/undischarged state, **(B)** first discharged to 1.1 V vs. Li/Li^+^ state, **(C)** first discharged to 0.6 V vs. Li/Li^+^ state. **(D–F)** The corresponding relationship between Zr and negative square root of angular frequency (ω^−1/2^) at various states. **(G–I)** The corresponding Li ion diffusion coefficient (*D*_*Li*_+) of MoS_2_ samples at various states.

To confirm the difference in electrochemical performance of the differentially expressed MoS_2_, EIS tests are performed to analyze the electronic conductivity and ion diffusion rate of the samples. Figure 6A shows the Nyquist plots at fully uncharged-undischarged state, accompanied by fitted equivalent circuit. The semicircular loop at the high-middle frequencies is related to the resistance of solid electrolyte interface and charge transfer resistance (R_ct_), while the slope line at low frequencies represents the Warburg impedance, which is connected to Li ion diffusion of the electrode materials(Wang et al., 2013a). The smaller semicircle of MoS_2_-1 μm compared with MoS_2_-5 μm, MoS_2_-2 μm, and MoS_2_-90 nm indicates a lower R_ct_. Thus, MoS_2_-1 μm is more conducive to charge transfer compared with the other samples (Jiang et al., 2017). Figure 6D shows the relationship between Z_r_ and negative square root of angular frequency (ω^−1/2^) in the low-frequency region at fully uncharged–undischarged state. Using the slope of the fitted line (Warburg coefficient), the Li ion diffusion coefficient can be calculated according to the following equation (Wu et al., 2016, 2017; Li et al., 2018):

(2) $$\begin{array}{l} D_{{Li}^{+}}=0.5R^{2}T^{2}/A^{2}n^{4}F^{4}C^{2}\sigma^{2} \end{array}$$

where *D*_*Li*_+ is the Li ion diffusion coefficient, *R* is the gas constant (8.314 J mol^−1^ K^−1^), *T* is the absolute temperature (298 K), *A* is the area of the electrode (1.53 cm^2^), *n* is the transfer electrons (for Li^+^, *n* = 1), *F* is the Faraday constant (96,485 C mol^−1^), C is the Li ion lattice concentration (0.001 mol cm^−2^), and σ is the Warburg coefficient. As shown in Figure 6G, at fully uncharged–undischarged state, the *D*_*Li*_+ values of MoS_2_-5 μm, MoS_2_-2 μm, MoS_2_-1 μm, and MoS_2_-90 nm are 7 × 10^−15^, 4.68 × 10^−15^, 7.56 × 10^−13^, and 3.21 × 10^−15^ cm^2^ s^−1^, respectively. Apparently, the Li ion diffusion coefficient of MoS_2_-1 μm is two orders of magnitude larger than those of the three other samples, which can reflect the higher initial capacity of MoS_2_-1μm (Figure 4C).

Figures 6B,E show the Nyquist plots at initially discharged to 1.1 V vs. Li/Li^+^ state where Li ion intercalation occurs and the corresponding relationship between Z_r_ and ω^−1/2^. The semicircles of MoS_2_-1 μm and MoS_2_-5 μm are smaller than those of MoS_2_-2 μm, and MoS_2_-90 nm, respectively. Thus, R_ct_ is lower and charge transfer is much easier for MoS_2_-1 μm and MoS_2_-5 μm than for MoS_2_-2 μm and MoS_2_-90 nm. The calculated *D*_*Li*_+ values of MoS_2_-5 μm, MoS_2_-2 μm, MoS_2_-1 μm, and MoS_2_-90 nm at initially discharged to 1.1 V vs. Li/Li^+^ state are 2.46 × 10^−13^, 2.19 × 10^−15^, 4.80 × 10^−13^, and 5.43 × 10^−14^ cm^2^ s^−1^, respectively (Figure 6H). On the basis of the results of R_ct_ and *D*_*Li*_+, the intensities of the Li ion intercalation can be ranked as MoS_2_-1 μm > MoS_2_-5 μm > MoS_2_-90 nm > MoS_2_-2 μm. As shown in Figure 6C, the semicircles of Nyquist plots, which at initially discharged to 0.6 V vs. Li/Li^+^ state where conversion reaction occurs, gradually enlarge from MoS_2_-1 μm to MoS_2_-5 μm. Simultaneously, the calculated *D*_*Li*_+ values for MoS_2_-5 μm, MoS_2_-2 μm, MoS_2_-1 μm, and MoS_2_-90 nm are 7.66 × 10^−14^, 1.9 × 10^−13^, 2.41 × 10^−12^, and 6.47 × 10^−13^ cm^2^ s^−1^, respectively (Figure 6I). These results indicate the significantly stronger conversion reactions of MoS_2_-1 μm than other samples.

**Figure 7:**
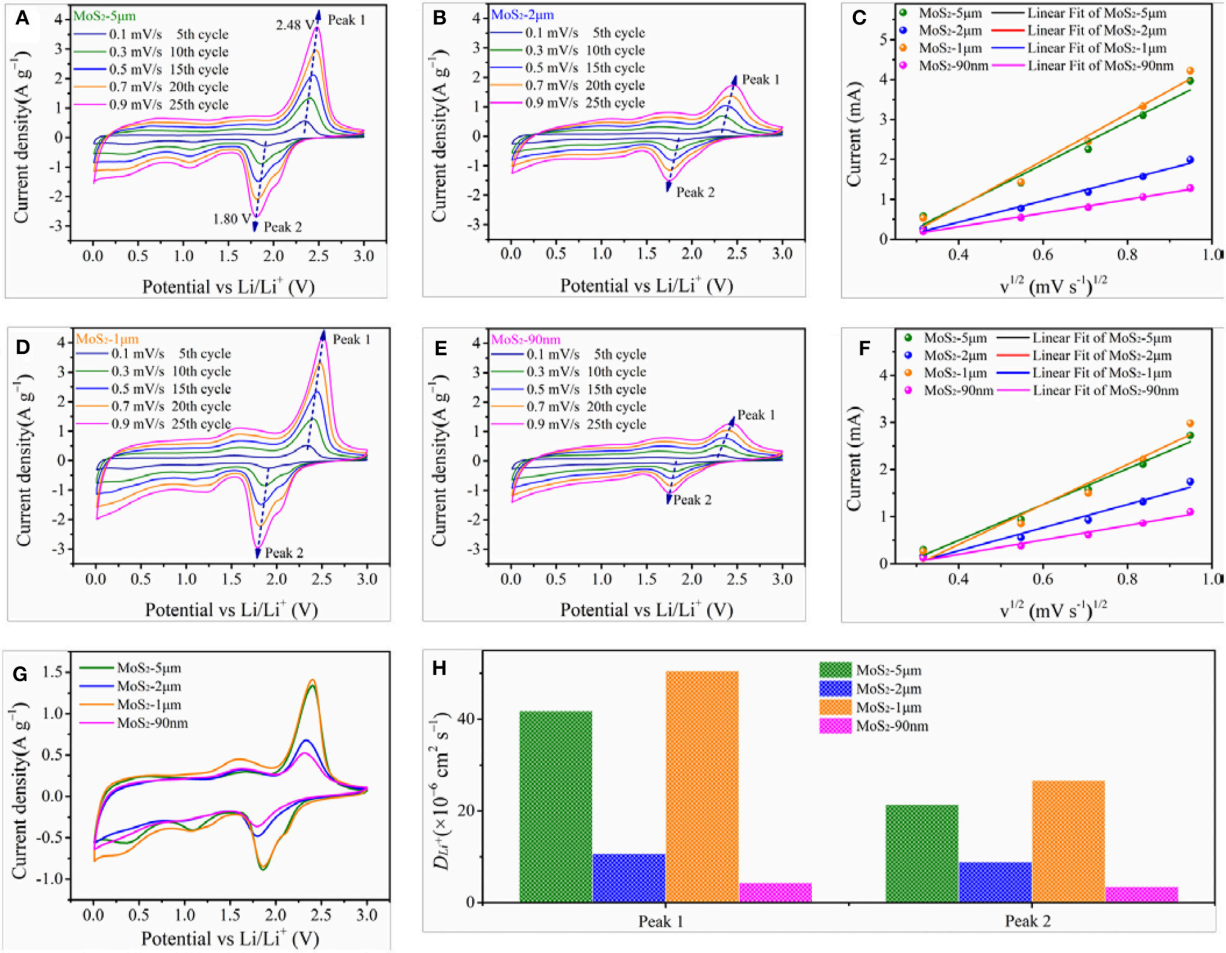
CV curves of **(A)** MoS_2_-5 μm, **(B)** MoS_2_-2 μm, **(D)** MoS_2_-1 μm and **(E)** MoS_2_-90 nm at various scanning rates from 0.1 to 0.9 mV s^−1^. The relationship between the peak current and square root of scan rate (v1/2) of **(C)** oxidation peak (Peak 1), **(F)** reduction peak (Peak 2). **(G)** Comparation of CV curves of MoS2 samples at 0.9 mV s^−1^. **(H)** Li ion diffusion coefficient (*D*_*Li*_+) of Peak 1 and Peak 2.

CV tests are conducted to further investigate the electrochemical kinetics of the as-prepared samples. Figures 7A,B,D,E show the CV curves of the MoS_2_ samples at different scanning rates, where the four MoS_2_ samples display similar CV behaviors. The dominant oxidation and reduction peaks appear at approximately 2.48 and 1.80 V vs. Li/Li^+^, respectively. Moreover, the peak at 2.48 V splits into two parts, which agrees well with the gradient conversion from element S_8_ to polysulfides and then to Li_2_S (Xiao et al., 2011). As the scan rate increasing, the peak current elevates, and the oxidation peak potential shifts positively while the reduction peak potential toward negatively. As shown in Figure 7G, the peak intensities clearly show the following trend: MoS_2_-1 μm > MoS_2_-5 μm > MoS_2_-90 nm > MoS_2_-2 μm, indicating the largest capacity of MoS_2_-1 μm electrode (Chou et al., 2011). Figures 7C,F show the relationship between the peak current and square root of scan rate (v^1/2^), which can be expressed by the following equation (Wang et al., 2013b; Sun et al., 2017):

(3) $$\begin{array}{l} i_{p}=2.69{\times 10^{5}n}^{\frac{3}{2}}AD^{\frac{1}{2}}v^{\frac{1}{2}}C_{0} \end{array}$$

where *i*_*p*_ is the peak current, *v* is the scan rate, *n* is the transfer electrons (for ${\text{Li}}_{,}^{+}$ *n* = 1), *A* is the area of the electrode (1.53 cm^2^), *D* is the Li ion diffusion coefficient, and Δ*C*_0_ is the change in Li^+^ concentration in the electrochemical reaction. Ion diffusion is a rate-determining step in the electrode. Thus, when scanning at a slow rate (<1 mV s^−1^), the peak current (*i*_*p*_) varied linearly with the square root of scan rate (*v*^1/2^). Hence, the slope can be utilized to characterize the Li ion diffusion coefficient (*D*). The results suggest that the fitting line slope of MoS_2_-1 μm is higher than that of the other samples (Figure 7H), revealing that MoS_2_-1 μm has better Li ion diffusion rate than the other samples, which is in good accordance with the EIS test results.

## Conclusions

Herein, 2D MoS_2_ sheets were successfully prepared from abundant natural raw molybdenite ore by a low-cost, environmental-friendly and high-yielding hydro-refining technology, containing crushing–grinding, flotation, physical exfoliation, and gradient centrifugation. Furthermore, the efficient tailoring and classification processes realized a series of size-controlled (5μm, 2μm, 1μm, 90nm) MoS_2_ sheets to improve Li-capacity and stability. When used as LIB anodes, size displayed significant effects on electrochemical performance. The MoS_2_-1 μm electrode demonstrated a higher initial charge capacity of 904 mAh g^−1^, further increasing to 1,337 mAh g^−1^ over 125 cycles at 0.1 A g^−1^. The excellent rate performance of the MoS_2_-1 μm electrode showed considerable capacities of 857 and 682 mAh g^−1^ at 2.0 and 5.0 A g^−1^, respectively. Owing to extraordinary morphology brought from tailoring craft, the as-prepared sheets offering rich active sites and defects for interacting with Li ions. Meanwhile, flexible structure could relieve volume expansion, significantly promoting the cycling stability. What's more, in-depth electrochemical kinetic analysis disclosed that the MoS_2_-1 μm electrode shows a lower charge transfer resistance and higher Li ion diffusion coefficient at various states, resulted from the successful size-tuning process. This work presents the remarkable effect of different MoS_2_ sheet sizes on Li-storage performance and provides a promising strategy for the large-scale production of MoS_2_-based LIB anodes from natural molybdenite mineral.

## Author contributions

FJ conducted the experiments. WS and XJ are the supervisor of this research work. SL, PG, and SK helped writing. HT and HH helped operating experiments. FJ, SL, HT, CZ, YY and YH performed the characterization and data analysis. All authors involved the analysis of experimental data and manuscript preparation.

### Conflict of interest statement

The authors declare that the research was conducted in the absence of any commercial or financial relationships that could be construed as a potential conflict of interest.
