# Supplementary material for: Size-Tunable Natural Mineral-Molybdenite for Lithium-Ion Batteries Toward: Enhanced Storage Capacity and Quicken Ions Transferring
Source: Front Chem. 2018 Aug 28;6:389. doi: 10.3389/fchem.2018.00389 (PMC6121191; doi:10.3389/fchem.2018.00389)
Supplement: Supplementary file 1 [file Table_1.DOCX]

Supporting Information


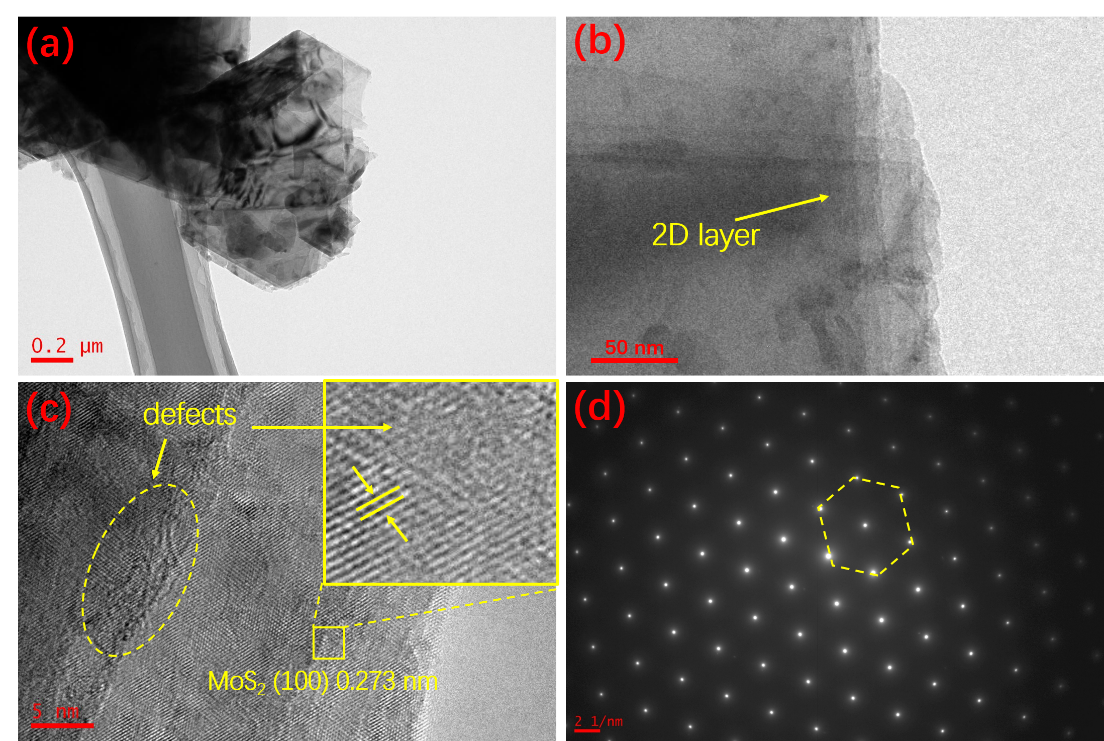


**Figure S1.** (a, b) TEM images; (c) HRTEM images; and (d) single-crystal SAED pattern of MoS_2_-1μm.


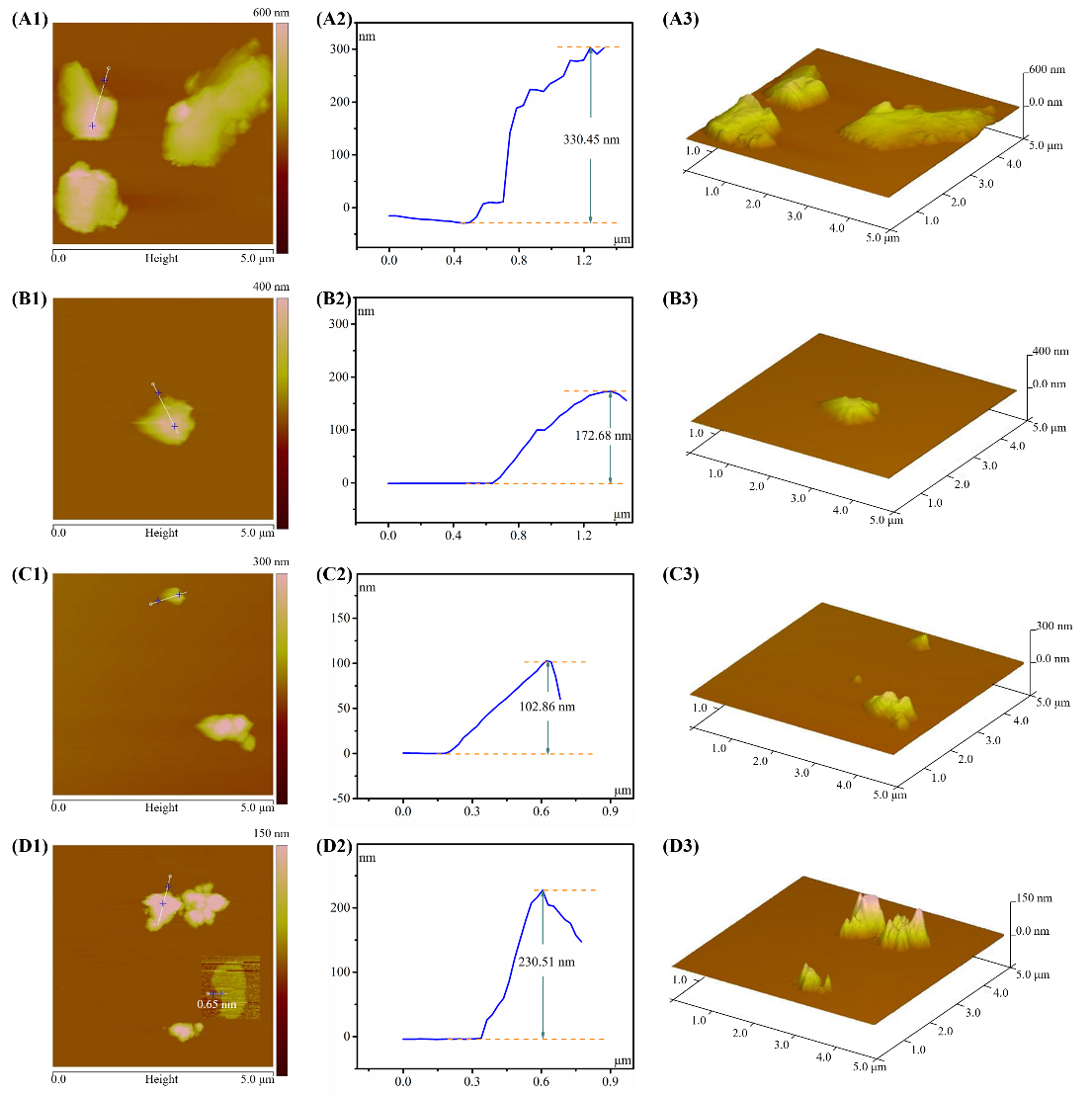


**Figure S2.** AFM images of (A1, A3) MoS_2_-5μm, (B1, B3) MoS_2_-2μm, (C1, C3) MoS_2_-1μm and (D1, D3) MoS_2_-90nm. Thickness of (A2) MoS_2_-5μm, (B2) MoS_2_-2μm, (C2) MoS_2_-1μm and (D2) MoS_2_-90nm sheets.
